# The Prevalence and Factors for Cancer Screening Behavior among People with Severe Mental Illness in Hong Kong

**DOI:** 10.1371/journal.pone.0107237

**Published:** 2014-09-30

**Authors:** Phoenix Kit Han Mo, Winnie Wing Sze Mak, Eddie Siu Kwan Chong, Hanyang Shen, Rebecca Yuen Man Cheung

**Affiliations:** 1 Center for Health Behaviours Research, Jockey Club School of Public Health and Primary Care, The Chinese University of Hong Kong, Shatin, New Territories, Hong Kong; 2 Department of Psychology, The Chinese University of Hong Kong, Shatin, New Territories, Hong Kong; 3 Department of Psychology, University of Maryland, College Park, College Park, Maryland, United States of America; 4 School of Public Health, Drexel University, Philadelphia, Pennsylvania, United States of America; 5 Department of Special Education and Counselling, Hong Kong Institute of Education, Tai Po, New Territories, Hong Kong; The Ohio State University, United States of America

## Abstract

**Objectives:**

Screening is useful in reducing cancer incidence and mortality. People with severe mental illness (PSMI) are vulnerable to cancer as they are exposed to higher levels of cancer risks. Little is known about PSMI's cancer screening behavior and associated factors. The present study examined the utilization of breast, cervical, prostate, and colorectal cancer screening among PSMI in Hong Kong and to identify factors associated with their screening behaviors.

**Method:**

591 PSMI from community mental health services completed a cross-sectional survey.

**Results:**

The percentage of cancer screening behavior among those who met the criteria for particular screening recommendation was as follows: 20.8% for mammography; 36.5% for clinical breast examination (CBE); 40.5% for pap-smear test; 12.8% for prostate examination; and 21.6% for colorectal cancer screening. Results from logistic regression analyses showed that marital status was a significant factor for mammography, CBE, and pap-smear test; belief that cancer can be healed if found early was a significant factor for pap-smear test and colorectal screening; belief that one can have cancer without having symptoms was a significant factor for CBE and pap-smear test; belief that one will have a higher risk if a family member has had cancer was a significant factor for CBE; and self-efficacy was a significant factor for CBE and pap-smear test behavior.

**Conclusions:**

Cancer screening utilization among PSMI in Hong Kong is low. Beliefs about cancer and self-efficacy are associated with cancer screening behavior. Health care professionals should improve the knowledge and remove the misconceptions about cancer among PSMI; self-efficacy should also be promoted.

## Introduction

### Cancer as an important health issue

Cancer is an important preventable cause of morbidity and mortality. It is a leading cause of death in Hong Kong, accounting for 30.6% of all deaths in 2010 [1]. Colorectal, prostate, breast, and cervical cancer are important public health problems in Hong Kong, accounting for 16.6% of total cancer incidence, 10.7% of cancer incidence in men, and 24.1% and 3.2% of cancer incidence in women in 2010 respectively. In addition, the number of invasive breast cancer and cervical cancer has increased by 2.3% and 26% in Hong Kong in 2009 respectively. Colorectal cancer is the second most common cancer for both sexes, and is projected to be the most common cancer in Hong Kong in the near future.

### Higher cancer risk among people with severe mental illness

People with severe mental illness (PSMI) are a vulnerable group to cancer as they are exposed to several factors associated with cancer, including poor lifestyle [2] such as heavy smoking [3], lack of exercise, poor diet [4], [5], and less than optimal physical health care [6], [7]. Indeed, studies have documented that PSMI tend to have higher level of cancer risk and cancer incidence, especially breast cancer and colorectal cancer, compared to the general population [8]–[12].

### Screening and cancer risk

There is established evidence that screening is effective in reducing incidence and mortality from cancer [13], [14]. In recent years, much attention has been given to the promotion of screening programs worldwide. Countries with mass screening programs for breast, cervical, prostate, and colorectal cancer have shown significant reductions in cancer incidence and mortality [15]–[18]. A strong correlation between screening and reduction in cost has been documented in many studies. For example, one study about cost effectiveness of cervical cancer screening in Hong Kong reported that a mass population-based cervical screening every 3 to 5 years for all women over aged 21 can substantially increase benefits and reduce cost compared with opportunistic screening in Hong Kong [19], supporting the importance of cancer screening for the general public.

### Cancer screening behavior in Hong Kong

Despite the effectiveness of the screening services, at present there are no centralized, systematic, population-based cancer screening programs in Hong Kong [20]. Most screening services are offered on an opportunistic basis [21]. Where a population-based approach screening is not available, research reported that the cancer screening utilization rate in Hong Kong is low and that misconception about cancer screening is widespread. For example, in a study of 430 women (87% aged 31–50 years) attendees of a women clinic in Hong Kong showed that 59% reported having a pap-smear test and 28% reported having a mammogram [22], such figures are lower than those reported in women of similar age range in other countries (i.e. 79.9% of those aged 25 or above reported recent pap-smear test and 66.9% of those above 40 years of age reported recent use of mammography in the United States) [23]. Similarly, population-based studies on colorectal cancer screening found that the uptake rate of any kind of colorectal cancer screening ranged from only 1.2%–4.5% (aged 18+) [24] to 9.9% (aged 30 to 65) [25] in Hong Kong. Another study on 1,664 clinic attendees aged 50 to 74 reported that 35% had taken fecal occult blood test [26]. One third (30.4%) had the wrong impression that they did not require colorectal cancer screening because they were asymptomatic [24].

In addition, PSMI have lower levels of cancer screening behavior compared to the general population [27], [28]. A recent narrative review found that overall, there is a 20% to 30% reduced likelihood of breast, cervical, and colorectal cancer screening in PSMI compared with those without severe mental illness [29]. Other studies also showed that women with mental illness are more than 81% less likely to receive adequate pap-smear screening compared with the general population despite their increased rates of smoking and increased number of primary care visits [30]. Additionally, they were found to be less adherent to breast cancer screening compared to those without mental illness (adjusted odds ratio = 0.60) [27]. Thus, there is an urgent need to promote cancer screening behavior among PSMI.

### Factors for cancer screening behavior

Research has identified various factors associated with the uptake of cancer screening. Studies in the Chinese population have reported lower education level, a paucity of screening information, access barriers such as lacking time and money [25],[31], and cognitive factors such as perceived barriers to screening [25] as factors for non-attendance to cancer screening services. In contrast, recommendations by doctors or nurses [25], [32], previous screening experience [22], family history of cancer [24], [26], knowledge about cancer [26], [33], and cognitive factors such as believing that cancer is potentially curable at an early stage [26], being concerned about cancer [24], and being conscious about health [22] have been identified as facilitators for cancer screening behavior. Interestingly, it has also been suggested that perceived severity of cancer is associated with a lower level of colorectal screening uptake among Chinese [25].

Furthermore, studies among PSMI have also found that family history of cancer and doctors' recommendations and referrals were key facilitators for mammogram screening uptake [34], [35]. From our understanding, no study examined the factors associated with cancer screening behavior among PSMI in the Chinese context.

### The present study

Despite the importance of cancer screening to the general public, very little is known about the cancer screening behavior of PSMI in Hong Kong and the factors associated with cancer screening behavior in this population. The present study aimed to examine the utilization of breast, cervical, prostate, and colorectal cancer screening among Chinese PSMI in Hong Kong, and to identify the factors associated with such screening behaviors. It is expected that findings would have important implications to guide the health care professionals to promote cancer screening among PSMI.

## Materials and Methods

### Study design

Stratified sampling based on gender, age, and diagnostic composition was used and PSMI were recruited from the various community mental health services in Hong Kong. These services represented the vast majority of the community support services, day training, and vocational rehabilitation services that are being provided to PSMI in Hong Kong. Inclusion criteria included: (1) age of 18 years or above, (2) residents of Hong Kong, (3) being able to understand Cantonese, which is the native spoken language in Hong Kong, (4) having at least one DSM-IV-TR Axis I diagnosis, (5) currently living in the community. Exclusion criteria included: (1) pregnancy or lactation, (2) intellectual disability, (3) dementia, (4) low levels of comprehension and cooperation.

### Procedure

Individual structured questionnaire was administered to the participants at one of the community mental health service centers. Each PSMI was interviewed by a trained interviewer on the structured questionnaire. The interview lasted for about 45 minutes. Participants were given a HK$70 ( = US$9.03) supermarket coupon as compensation for their time spent in the study.

### Ethics Statement

Written consent was obtained from each participant before the survey began. Staff of the community mental health services assessed participants' cognitive ability and participants were excluded if they were found to have low levels of comprehension which precluded their ability to provide consent. Separate written consent was also obtained from the participants for their diagnoses, health check records, and medications from their medical records. The consent forms were stored separately with participants' questionnaire in a locked cabinet and were only accessible to the research team. The consent and study procedure was approved by the Survey and Behavioral Ethics Committee of the Chinese University of Hong Kong.

### Measures

#### Demographics

Participants' gender, age, education level, living condition, employment status, financial situation, and diagnoses (physical and psychiatric) were obtained. Where available, participants' diagnoses and medications were verified with their medical records upon consent.

#### Knowledge and perceptions about cancer

Items on knowledge and perceptions about cancer from the Health Risk factors Questionnaire were used to measure participants' knowledge and perception about cancer [36]. The Questionnaire was developed for the Asian American Health Needs Assessment (AsANA). Its Chinese version has been tested and considered culturally tailored and linguistically appropriate.

#### Self-efficacy

One item was used to assess participants' self-efficacy in performing body health check: “How confident are you in performing body health check regularly?” The item was rated on a 4-point Likert scale from (1) *not at all* to (4) *definitely*, with higher scores indicating greater self-efficacy.

#### Cancer screening behavior

Items on cancer screening from the Health Risk Factors Questionnaire were used to assess participants' cancer screening behavior [36]. Five types of cancer screening were assessed, based on the screening guideline from the American Cancer Society [37], participants who met the criteria for recommendation on a particular cancer screening were asked if they have ever taken such screening: (1) mammography (for women aged 40 years or above); (2) clinical breast examination (CBE; for women aged 20 years or above); (3) pap-smear test (for women aged 21 to 65 years); (4) any kind of prostate examination (prostate specific antigen or digital rectal exam; for men aged 50 years or above); and (5) any kind of colorectal screening (fecal occult blood test, flexible sigmoidoscopy, colonoscopy, double-contrast barium enema, or computed tomography colonography; for men and women aged 50 years or above).

### Data analysis

Descriptive analyses were performed. To identify factors for the uptake of various types of cancer screening, univariate odds ratio (OR) and respective 95% confidence intervals were examined by fitting logistic regression models. Multivariate forward stepwise logistic regression analyses were then performed to obtain a summary model and those independent variables with p<.05 in the univariate analysis were used as candidates for the forward stepwise selection procedure, which stopped when the entry of any additional variable brought no further improvement to the model's fitness. A subset of significant factors was hence identified. Data analysis was performed by using SPSS 18.0 for Windows.

## Results

### Background characteristics of the participants

A total of 591 participants took part in the survey. Slightly more than half (54.0%) of the participants were female. A majority of them (73.6%) were over 40 years old. Respectively, 48.7%, 22.3%, 6.0%, and 23.0% described their primary mental illness diagnosis as schizophrenia, depression, bipolar disorder, and others. Their mean duration of mental illness was 17.7 years (SD = 10.9) (Table 1).

**Table 1:** Background Characteristics of the Participants.

Background characteristics	Total (n = 591)#
Gender	
Male	266 (45.0%)
Female	325 (54.0%)
Age	
<20	5 (0.9%)
20–30	33 (5.8%)
30–40	113 (19.7%)
40–50	183 (31.9%)
50–60	185 (32.3%)
60–70	49 (8.6%)
>70	5 (0.8%)
Education level	
Primary School or below	139 (23.6%)
Secondary School	405 (68.9%)
College or above	44 (7.5%)
Marital Status	
Single	320 (54.3%)
Married/Cohabited	133 (22.6%)
Divorced/Separated/Widowed	136 (23.1%)
Employment Status	
Unemployed	195 (33.1%)
Employed	394 (66.9%)
Monthly income	
HKD4000 or below	456 (79.2%)
HKD4000–8000	109 (18.9%)
HKD8000 above	11 (1.9%)
Type of mental illness	
Schizophrenia	286 (48.7%)
Depression	131 (22.3%)
Bipolar Disorder	35 (6.0%)
Others	135 (23.0%)
Duration of mental illness (in years)	M = 17.7, SD = 10.9

# Total number varied slightly for each variable due to missing data.

### Cancer screening behavior among participants

Approximately one-fifth (20.8%) of women over 40 years old reported ever having a mammography screening; one third of these women (34.7%) reported having ever had screening in the past year. Slightly more than one third (36.5%) of women over 20 years old reported ever having a CBE; with 41.6% of them reported having had one in the past year. More than one-third (40.5%) of women aged 21 to 65 years reported ever having had a pap-smear test; with 39.8% of them reported having had a test in the past year. Next, 12.8% of men aged 50 or above reported ever having had a prostate examination. One-fifth (21.6%) of participants aged 50 or above reported having ever had a colorectal cancer screening. Among them, 29.9% have taken a fecal occult blood test; 17.2% have taken flexible sigmoidoscopy; 34.5% have taken colonoscopy; and 18.4% have taken double contrast barium enema (Table 2). The cancer screening rate was lower than those reported in other local or international studies (Table 3).

**Table 2:** Cancer Screening Behavior among People with Severe Mental Illness.

Cancer screening behavior	Total
Mammography screening (Women aged ≥40; n = 236)^1^	
Ever had a screening	49 (20.8%)
Never had a screening	187 (79.3%)
How long since the last time mammography? (Among those who had mammography; n = 49)	
Within 1 year (≤1 year)	17 (34.7%)
Within 2 years (>1 year but ≤2 years)	13 (26.5%)
Within 3 years (>2 years but ≤3 years)	6 (12.2%)
Within 5 years (>3 years but ≤5 years)	3 (6.1%)
Over 5 years	9 (18.4%)
Don't know/remember	1 (2.0%)
Clinical breast examination (CBE) (Women aged ≥20; n = 310)^1^	
Ever had a screening	113 (36.5%)
Never had a screening	197 (63.5%)
How long since the last time CBE? (Among those who had CBE; n = 113)	
Within 1 year (≤1 year)	47 (41.6%)
Within 2 years (>1 year but≤2 years)	16 (14.2)
Within 3 years (>2 years but≤3 years)	13 (11.5%)
Within 5 years (>3 years but≤5 years)	14 (12.4%)
Over 5 years	22 (19.5%)
Don't know/remember	1 (0.9%)
Pap-smear Test (Women aged 21 to 65; n = 304)^1^	
Ever had a screening	123 (40.5%)
Never had a screening	181 (59.5%)
How long since the last time Pap-smear test? (Among those who had pap-smear test; n = 123)	
Within 1 year (< = 1 year)	49 (39.8%)
Within 2 years (>1 year but ≤2 years)	26 (21.1%)
Within 3 years (>2 years but ≤3 years)	14 (11.4%)
Within 5 years (>3 years but ≤5 years)	16 (13.0%)
Over 5 years	18 (14.6%)
Prostate Examination (Men aged ≥50; n = 109)^1^	
Ever had a screening	14 (12.8%)
Never had a screening	95 (87.1%)
Colorectal cancer screening (Men and women aged ≥50; n = 236)^1^	
Ever had a screening	51 (21.6%)
Never had a screening	185 (78.4%)
Type of colorectal cancer screening taken (Among those who had colorectal cancer screening; n = 87)^2^	
Fecal occult blood test	26 (29.9%)
Flexible sigmoidoscopy	15 (17.2%)
Colonoscopy	30 (34.5%)
Double contrast barium enema	16 (18.4%)

1 The age for each cancer screening was set based on the screening guidelines of the American Cancer Society.

2 Total number higher than the number of people having taken colorectal cancer screening as some participants have taken more than one type of colorectal cancer screening.

**Table 3:** Cancer Screening Behavior Reported in Other Studies.

**Mammography**						
Study	Current study	Abdullah et al. [22]	Kwok et al. [41]	Kwok et al. [42]	Ma et al. [44]	Breen et al. [23]
Country	Hong Kong	Hong Kong	Hong Kong	Australia	United States	United States
Targeted population	Chinese women with MI ≥40 years	Chinese women aged 31–50 years	Chinese women aged 50–69 years	Chinese-Australian women aged 50–69 years	Chinese-American women ≥40 years	Women ≥40 years
Sample size	236	430	150	104	NA	10,374
Screening rate	20.8%	28%	32.7%	75.0%	79.9%	66.9%
Time frame	Ever	Ever	Every two years	Every two years	Ever	Last 2 years
**Clinical Breast Examination**						
Study	Current study	Kwok et al. [41]	Tang et al. [51]	Kwok et al. [42]		
Country	Hong Kong	Hong Kong	United States	Australia		
Targeted population	Chinese women with MI ≥20 years	Chinese women ≥40 years	Chinese-American women ≥60 years	Chinese-Australian women ≥40 years		
Sample size	310	320	100	158		
Screening rate	36.5%	37.8%	70%	35.4%		
Time frame	Ever	Annually	Ever	Annually		
**Pap-smear Test**						
Study	Current study	Abdullah et al. [22]	Ma et al. [44]	Breen et al. [23]		
Country	Hong Kong	Hong Kong	United States	United States		
Targeted population	Chinese women with MI aged 21–65 years	Chinese women aged 31–50 years	Chinese-American women ≥18 years	Women ≥25 years		
Sample size	304	430	NA	15,704		
Screening rate	40.5%	59%	72.1%	79.9%		
Time frame	Ever	Ever	Ever	Last 3 years		
**Prostate Examination**						
Study	Current study	Ma et al., [43], [44]	Breen et al. [23] *	McKinley et al. [52]		
Country	Hong Kong	United States	United States	Australia		
Targeted population	Chinese men with MI ≥50 years	Chinese-American men ≥50 years	Men ≥50 years	Male BRCA1 and BRCA2 carriers aged 40–69 years		
Sample size	109	163	4,871	75		
Screening rate	12.8%	43.3%	50.0%	55% for PSA, 43% for DRE		
Time frame	Ever	Ever	Last 2 years	Last 3 years		
**Colorectal Cancer Screening**						
Study	Current study	Sung et al. [25]	Ma et al. [44]	Breen et al. [23]		
Country	Hong Kong	Hong Kong	United States	United States		
Targeted population	Chinese men and women with MI ≥50 years	Chinese men and women aged 30–65 years	Chinese American men and women ≥50 years	Women ≥50 years		
Sample size	236	1,004	NA	11,679		
Screening rate	21.6%	9.9%	34.7%	30.2% for women, 37.1% for men		
Time frame	Ever	Ever	Ever	Last 3 years		

* Only digital rectal examination was reported in the study

Abbreviations: MI = Mental illness; PSA = Prostate specific antigen test; DRE = Digital rectal examination; NA = Data not available

### Knowledge and perceptions about cancer

A majority of the participants (81.6%) agreed that cancer could be healed if found early, two third of them (67.5%) believed one could have cancer without having symptoms. Most of them (90.6%) perceived some chances of getting cancer, and about two third (63.1%) believed that they would have a higher risk of developing cancer if a family member has had cancer. Half of them (50.1%) did not know whether they could find materials and services related to cancer (Table 4).

**Table 4:** Knowledge and Perceptions about Cancer among People with Severe Mental Illness.

Knowledge and perceptions	Total (%)
Do you think cancer in general can be cured if detected early?	
Yes	482 (81.6%)
No/don't know	109 (18.4%)
Do you know where you can get information on cancer or cancer services?	
Yes	295 (49.9%)
No/don't know	296 (50.1%)
Do you think cancer is caused by fate or higher power?	
Yes	214 (36.2%)
No	377 (63.8%)
Do you think that you can have cancer but not have symptoms?	
Yes	399 (67.5%)
No/don't know	192 (32.5%)
What do you think your risks are for developing cancer?	
Not at risk	50 (9.4%)
Low/moderate risk	401 (75.5%)
High risk	80 (15.1%)
Do you think having a family member who has had cancer increases your risk of developing cancer?	
Yes	372 (63.1%)
No/don't know	218 (36.9%)

### Factors related to mammography behavior among women aged 40 years or above

Results from the univariate logistic regressions showed that among all the variables, only marital status was significant in predicting mammography behavior, with those who were married or cohabitated being more likely to reported ever having had a mammography (OR = 2.69, 95% CI = 1.19, 6.07) (Table 5).

**Table 5:** Logistic Regression on Cancer Screening Behavior among People with Severe Mental Illness.

	Mammography			Clinical Breast Examination			Pap-smear Test			Prostate Examination			Colorectal Cancer Screening		
	(Female aged ≥40) (N = 236)			(Female aged ≥20) (N = 310)			(Female aged 21 to 65) (N = 314)			(Male aged ≥50) (N = 109)			(All aged ≥50) (N = 236)		
	Row%	OR_U_ (95%CI)	OR_M_ (95%CI)	Row%	OR_U_ (95%CI)	OR_M_ (95%CI)	Row%	OR_U_ (95%CI)	OR_M_ (95%CI)	Row%	OR_U_ (95%CI)	OR_M_ (95%CI)	Row%	OR_U_ (95%CI)	OR_M_ (95%CI)
**Age**	—	1.02(.97–1.07)	—	—	1.01(0.99–1.03)	—	—	1.01(.99–1.04)	—	—	1.04(.95–1.13)	—	—	**1.06(1.00**–**1.12)** *	—
**Gender**															
Male	—	—	—	—	—	—	—	—	—	—	—	—	21.3	1	—
Female													21.5	1.010.55–1.89)	
**Education level**															
Primary School or below	15.0	1	—	32.9	1	—	49.4	1	—	11.4	1	—	20.2	1	—
Secondary School	25.0	1.89(.92–3.89)		36.0	1.15(.67–1.96)		39.3	.66(.40–1.11)	.90(.48–1.66)	14.7	1.34(.39–4.61)		22.0	1.12(.59–2.13)	
College or above	13.3	0.87(.17–4.36)		54.2	2.41(.96–6.05)		**20.8**	**.27(.09**–**.79)** *	.41(.12–1.38)	0	—		27.3	1.48(.36–6.10)	
**Employment Status**															
Unemployed	23.0	1	—	35.7	1	—	45.0	1	—	13.5	1	—	23.3	1	—
Employed	19.5	0.81(.43–1.54)		36.9	1.05(.65–1.70)		37.9	.75(.47–1.21)		12.7	.93(.29–3.00)		20.4	.85(.45–1.60)	
**Marital Status**															
Single	14.1	1	—	27.1	1	—	19.7	1	—	12.1	1	—	20.4	1	—
Married/Cohabited	30.6	**2.69(1.19**–**6.07)** **		46.2	**2.30(1.28**–**4.16)** **	**2.22(1.14**–**4.33)** *	58.4	**5.74(3.06**–**10.77)** ***	**5.22(2.57**–**10.6)** ***	14.3	1.21(.32–4.55)		20.9	1.03(.47–2.23)	
Divorced/Separated/Widowed	20.2	1.54(.69–3.43)		40.8	**1.85(1.06**–**3.21)** *	1.58(.84–2.97)	53.0	**4.60(2.56**–**8.28)** ***	**3.80(1.96**–**7.36)** ***	13.6	1.15(.27–4.91)		23.1	1.17(.56–2.42)	
**Monthly Income**															
$4000 or below	19.6	1		33.6	1	—	36.7	1	—	11.8	1	—	22.1	1	—
$4000–$8000	27.5	1.56(.71–3.42)	—	49.2	**1.91(1.08**–**3.38)** *	1.64(.87–3.12)	**54.1**	**2.03(1.15**–**3.60)** *	1.50(.78–2.85)	20.0	1.88(.52–6.74)		25.0	1.18(.51–2.69)	
$8000 above	16.7	.82(.09–7.26)	—	42.9	1.48(.32–6.78)	1.37(.20–9.39)	71.4	4.32(.82–22.74)	3.21(.54–19.08)	0	—	—	0	—	
**Mental Illness type**															
Schizophrenia	16.7	1	—	32.0	1	—	39.7	1	—	9.1	1	—	12.4	1	—
Depression	27.3	1.88(.90–3.91)		43.2	1.62(.93–2.82)		50.0	1.52(.88–2.63)		11.8	1.33(.23–7.58)		31.3	**3.23(1.46**–**7.16)** **	**3.42(1.52**–**7.70)** **
Bipolar Disorder	17.6	1.07(.28–4.16)		48.0	1.96(.82–4.70)		40.0	1.01(.42–2.44)		33.3	5.00(.38–65.36)		30.0	3.04(.69–13.36)	**3.71(1.01**–**17.07)** *
Others	19.6	1.22(.49–3.01)		30.9	.95(.50–1.80)		28.8	.62(.32–1.17)		15.6	1.85(.49–6.97)		23.8	2.21(.96–5.12)	**2.36(1.00**–**5.55)** *
**Do you think cancer in general can be cured if detected early?**															
No/don't know	10.9	1	—	22.8	1	1	28.1	1	—	21.1	1	—	8.9	1	1
Yes	23.2	2.47(.92–6.64)		39.5	**2.21(1.13**–**4.32)** *	1.54(.72–3.27)	43.3	**1.96(1.04**–**3.68)** *	**2.00(1.01**–**4.13)** *	11.1	.47(.13–1.69)		24.2	**3.28(1.12**–**9.63)** *	**3.49(1.17**–**10.43)** **
**Do you know where you can get information on cancer or cancer services?**															
No/don't know	22.9	1	—	32.7	1	—	41.6	1	—	10.2	1	—	19.7	1	—
Yes	18.1	.74(.39–1.41)		40.4	1.40(.88–2.22)		39.3	.91(.58–1.44)		16.0	1.68(.54–5.23)		23.5	1.25(.67–2.33)	
**Do you think cancer is caused by fate or higher power?**															
No/don't know	17.7	1	—	39.0	1	—	44.2	1	—	9.1	1	—	21.1	1	—
Yes	25.3	1.57(.83–2.95)		32.2.74(.46–1.21)			34.2	.66(.41–1.06)		18.6	2.29(.73–7.13)		21.6	1.03(.55–1.94)	
**Do you think that you can have cancer but not have symptoms?**															
No	14.9	1	—	17.7	1	—	21.1	1	—	11.1	1	—	18.3	1	—
Yes	23.5	1.76(.84–3.67)		44.9	**3.78(2.10**–**6.82)** ***	**3.12(1.62**–**6.02)** ***	49.3	**3.64(2.08**–**6.40)** ***	**2.81(1.49**–**5.29)** ***	13.7	1.27(.37–4.37)		22.9	1.33(.68–2.60)	
**What do you think your risks are for developing cancer?**															
Not at risk	14.3	1	—	18.5	1	—	37.0	1	—	0.0	—	—	17.6	1	—
															
Low/moderate risk	19.2	1.43(.39–5.17)		40.0	**2.93(1.07**–**8.06)** **	2.18(.73–6.49)	39.8	1.12(0.49–2.58)		12.0			21.9	1.31(.36–4.80)	
High risk	32.4	2.88(.71–11.71)		41.7	**3.14(1.02**–**9.71)** ***	2.08(.61–7.11)	45.8	1.44(0.55–3.78)		23.1			24.2	1.49(.34–6.56)	
**Do you think having a family member who has had cancer increases your risk of developing cancer?**															
No/don't know	20.3	1	—	25.0	1	—	30.2	1	—	5.7	1	—	18.1	1	—
Yes	21.0	1.05(.54–2.05)		42.1	**2.18(1.29**–**3.71)** **	1.48(.79–2.77)	45.4	**1.92(1.15**–**3.22))** *	**1.65(1.02**–**3.04)** *	18.2	3.70(.96–14.31)		24.1	1.43(.76–2.71)	
**Self-efficacy**	—	1.28 (.95,1.75)	—	—	**1.34 (1.12**–**1.64)** *	**1.17 (1.03**–**1.54)** *	—	**1.69 (1.31**–**2.20)** *	**1.55(1.16**–**2.07)** *	—	1.16 (.75–1.79)		—	1.26 (.90–1.77)	

*p<.05,

**p<.01,

***p<.001, Abbreviations: OR_U_ = odds ratio obtained using univariate logistic regression, OR_M_ = odds ratio obtained from stepwise multivariate logistic regression analysis using univariately significant variables as candidate variables; CI = confidence interval;

— not applicable.

### Factors related to CBE behavior among women aged 20 years or above

Results from the univariate logistic regressions showed that among all the variables, marital status, monthly income, belief that cancer can be healed if found early, belief that one can have cancer without having symptoms, belief that their risk of getting cancer is high, belief that they will have a higher risk if a family member has had cancer, and self-efficacy were significant predictors to CBE behavior. These variables were selected and considered in the multivariate forward stepwise logistic regression procedures. Results of the multivariate stepwise analysis showed that those who were married or cohabited (OR = 2.22, 95% CI = 1.14, 4.33), believed that one can have cancer without having symptoms (OR = 3.12, 95% CI = 1.62, 6.02), and those who had a higher level of self-efficacy (OR = 1.17, 95% CI = 1.03, 1.54) were more likely to reported ever having had CBE (Table 5).

### Factors related to pap-smear test behavior among women aged 21 to 65 years

Results from the univariate logistic regressions showed that among all the variables, education level, marital status, monthly income, belief that cancer can be healed if found early, belief that one can have cancer without having symptoms, belief that they will have a higher risk if a family member has had cancer, and self-efficacy were significant predictors for pap-smear test behavior. In the multivariate stepwise analysis, not being single (OR = 5.22, 95% CI = 2.57, 10.6 for those who were married/cohabited and 3.80, 95% CI = 1.96, 7.36 for those who were divorced/separated/widowed), belief that cancer could be healed if found early (OR = 2.00, 95% CI = 1.01, 4.13), belief that one can have cancer without having symptoms (OR = 2.81, 95% CI = 1.49, 5.29), belief that they would have a higher risk if a family member has had cancer (OR = 1.65, 95% CI = 1.02, 3.04), and self-efficacy (OR = 1.55, 95% CI = 1.16, 2.07) were significant factors for having had pap-smear test (Table 5).

### Factors related to prostate examination behavior among men aged 50 years or over

Results from the univariate logistic regressions showed that none of the variables were significant predictor on prostate examination behavior (Table 5).

### Factors related to colorectal screening behavior among all participants aged 50 or above

Results from the univariate logistic regressions showed that among all the variables, mental illness type and belief that cancer can be healed if found early were significant predictors to colorectal screening behavior. These variables remained significant in the multivariate stepwise logistic regression analysis, with those who had diagnosis of depression (OR = 3.42, 95% CI = 1.52, 7.70), bipolar disorder (OR = 3.71, 95% CI = 1.01, 17.07) or other types of mental illness (OR = 2.36, 95% CI = 1.00, 5.55), and believed that cancer could be healed if found early (OR = 3.49, 95% CI = 1.17, 10.43) being more likely to report ever having had a colorectal screening (Table 5).

## Discussions

With its increasing incidence, cancer has become a major public health issue in Hong Kong. The study reported the pattern of cancer screening behavior, the knowledge and perceptions about cancer, and factors related to cancer screening behavior among Chinese PSMI in Hong Kong. Findings of the study showed that misconceptions about cancer were prevalent among PSMI. Specifically about one fifth did not think that cancer could be healed if found early, more than one third believed that fate was the cause of cancer, disagreed that one could have cancer without having symptoms, and disbelieved that one would have a higher risk if a family member has had cancer. This reflects a lack of health education about cancer among PSMI. The results were consistent to a previous study showing that cancer fatalism was evident among Chinese women in the United States, who believed that people had no control over life and death and therefore regular cancer screenings were not important [38]. Appropriate knowledge about cancer is fundamental in improving awareness of cancer, it also has the potential to increase one's motivation to take up cancer screening. There is a need to clarify misconceptions and to improve the awareness about cancer and cancer screening among PSMI.

One important point to note is that in addition to the misconceptions about cancer, barriers to obtain cancer-related information also seemed to be common among PSMI, with half of them not knowing where to find relevant materials and services. These barriers may explain PSMI's widespread misconceptions about cancer and corroborate the extant literature that demonstrated a high level of unmet needs in obtaining health information among PSMI [39]. Findings suggest that provision of health information to PSMI should not only focus on the mental illness diagnosis. Rather, information related to the most common physical morbidity, such as cancer, should be provided. Mental health professionals should also highlight resources that facilitate PSMI to seek further cancer-related services as well as information about the availability and effectiveness of different types of cancer screening.

Findings of the present study show that the uptake of cancer screening is low among Chinese PSMI. The low prevalence of cancer screening may be due to the lack of organized population-based screening program in Hong Kong. It also implies that the promotion and delivery of cancer screening services in Hong Kong is suboptimal. Indeed, certain cancer screening, such as mammography, has not been widely promoted in Hong Kong as there has been controversies that it may not be cost effective [40]. Furthermore, findings of the present study showed that the uptake of cancer screening of PSMI is generally lower than those of the Hong Kong general population [22], [25], [41] and those from other countries [23], [42]–[44]. A low rate of cancer screening among PSMI as observed in the current study is consistent with results from previous studies [27]–[30]. One possible explanation might be that for PSMI, the priority in treating mental illness might supersede other physical concerns such as cancer. In Hong Kong, a comprehensive health care for PSMI is insufficient. Treatment to PSMI has been heavily focused on their psychiatric condition whereas their physical health needs have been relatively ignored. A lack of motivation may also prevent PSMI from regularly seeking preventive health care [45]. Furthermore, given that a broadly based cognitive impairment has been observed among PSMI [46], PSMI might have more difficulty in understanding the screening procedure as well as its risks and benefits, which might eventually contribute to a low level of cancer screening uptake. These factors signify the importance of health care professionals working with PSMI to pay more attention in the physical health needs of PSMI, and to raise the awareness of PSMI in taking part in cancer screening as a part of preventive health practices.

Findings also suggested that knowledge and perceptions about cancer are strongly associated with cancer screening behavior. In the present study, the belief that cancer can be healed if found early was related to CBE, pap-smear screening, and colorectal cancer screening. The belief that one can have cancer without having symptoms was associated with CBE and pap-smear screening. The belief that one will have a higher risk if a family member has had cancer was related to pap-smear screening. It seems that PSMI who have taken cancer screening believed in its benefits in terms of its prevention nature. Knowledge about cancer also seems to be an important factor in the utilization of screening services among Chinese PSMI. To promote utilization of cancer screening among PSMI, information about cancer and the benefits of screening should be provided in easily understandable, accessible way. Tailor-made information should also be designed by addressing their beliefs and concerns, and modifying possible misconceptions about cancer. Interventions are warranted to help PSMI process information concerning cancer and cancer screening so that they recognize the importance and the benefits associated with cancer screening.

As suggested by the socio-cognitive theories of health, self-efficacy is important to the implementation and maintenance of a health behavior [47], [48]. In the present study, self-efficacy is related to CBE and pap-smear screening. The belief that a person holds about his/her ability to perform a health behavior can influence strongly on the power he/she has and the decisions he/she is likely to make with regards to the health behavior. Therefore, it may be possible that those with a higher level of self-efficacy believe in their ability to perform cancer screening and are more likely to view cancer screening as something to be undertaken rather than something to be avoided. Self-efficacy is strongly associated to increased participation in cancer screening programs for various populations [49], [50]. The present study confirmed such relationship among Chinese PSMI and suggested that self-efficacy on cancer screening should be promoted in this population.

### Limitations

The present study had several limitations. First, the participants were recruited from community mental health organizations and may not necessarily represent the cancer screening utilization pattern of the general PSMI population in Hong Kong. However it is important to note that a stratified sampling was used in recruiting participants. Second, the study was cross-sectional, thus the associations observed could not be assumed as causal. Longitudinal studies are warranted to establish the temporal validity of the associations obtained in the present study. Third, variables were measured using single item and the number of PSMI having taken up certain cancer screening (e.g. prostate cancer screening) was small, these may account for the non-significant relationship among the variables. It should nevertheless be noted that single item has been commonly used in research on screening behaviors [22], [24], [32], and the items used in the present study were from a validated measure. Fourth, cancer screening behaviors were self-reported using a retrospective design, therefore the utilization of various cancer screenings might be overestimated due to recall bias or social desirability. Future research should consider the use of objective assessments of cancer screening behavior in this population. Finally, the lack of a comparison group precluded the opportunity that a comparison of cancer screening rate of PSMI to other populations could be made. Although a summary of the cancer screening rate reported by different populations was included, such figures were not directly comparable due to the use of different research methodologies and different targeted populations. Future studies should seek to include a comparison group so as to allow direct comparison of cancer screening rate of PSMI to those of other populations.

### Conclusion

Cancer is a public health concern that is preventable by timely and regular cancer screening. The present study suggested that cancer screening utilization among PSMI in Hong Kong is low. Knowledge and perceptions about cancer and self-efficacy are important factors for cancer screening behavior. Findings suggest that in addition to emphasizing information and treatment about PSMI's mental health status, health care professionals also should seek to improve the knowledge and remove the misconceptions about cancer among PSMI. Self-efficacy should also be promoted so as to increase their cancer screening behavior.
